# Regulation of photosynthesis, carbohydrate transport, and transcriptomic profiles in wheat by different fertilizers

**DOI:** 10.1186/s12870-026-09506-6

**Published:** 2026-07-18

**Authors:** Yan Li, Xiaolin Zhou, Zishuang Li, Lei Ma, Jianlin Wei, Deshui Tan

**Affiliations:** 1 State Key Laboratory of Nutrient Use and Management, Ji’nan, China; 2 Institute of Agricultural Resources and Environment, Shandong Academy of Agricultural Sciences, Ji’nan, China; 3 Dezhou Academy of Agricultural Sciences, Dezhou, China

**Keywords:** Organic-inorganic compound fertilizer, Dry-hot wind, Photosynthetic gene, Non-structural carbohydrate transport, Carbohydrate accumulation

## Abstract

**Background:**

In winter wheat cultivation, the rational application of organic-inorganic compound fertilizers increases grain weight and improves yield; however, the underlying mechanisms remain unclear. A 3-year field trial was conducted to investigate the effects of three fertilization regimes on photosynthesis, carbohydrate transport, and gene expression in wheat. The treatments included chemical fertilizer alone (CK), chemical fertilizer supplemented with composted pig manure (T1), and organic-inorganic compound fertilizer (T2).

**Results:**

The T2 treatment resulted in significantly higher chlorophyll content and net photosynthetic rates in flag leaves compared to CK and T1. During the grain-filling stage, RuBP carboxylase activity in T2 increased significantly by 12.3% to 15.6%. Furthermore, the transport rate of non-structural carbohydrates (NSCs) in the stem sheaths of T2-treated wheat increased significantly by 23.84% and 20.38% compared to CK and T1, respectively. Correspondingly, NSC accumulation in the grains increased significantly by 15.80% and 18.94%, respectively. ADPG pyrophosphorylase activity in T2 was also significantly higher (by 72.4%) during the filling stage than in CK. Transcriptome analysis revealed that the highest number of differentially expressed genes (DEGs) associated with photosynthesis and carbon cycling were abundantly enriched in the flag leaves of T2. Specifically, in the T2 vs. T1 comparison, 1,420 up-regulated genes were primarily involved in photosynthesis and the carbon cycle, whereas in the T2 vs. CK comparison, 3,266 up-regulated genes were mainly associated with photosynthesis and cellular responses to light stimulation. Additionally, in the T2 treatment, several up-regulated genes involved in carbon fixation in photosynthetic organisms were simultaneously associated with the glyoxylate and dicarboxylate metabolism pathways. The most frequently identified DEGs in T2 belonged to the MYB-related, bZIP, ERF, and HB-other transcription factor (TF) families.

**Conclusion:**

The application of organic-inorganic compound fertilizers improves winter wheat yield by enhancing photosynthesis and the transport and accumulation of NSCs. Furthermore, it up-regulates the transcriptomic expression of genes related to photosynthesis and carbon cycling.

**Supplementary Information:**

The online version contains supplementary material available at https://doi.org/10.1186/s12870-026-09506-6.

## Introduction

Wheat is a cornerstone staple food for the global population. The winter wheat-summer maize rotation system is a widely adopted agricultural practice, particularly in regions such as the North China Plain. Developing wheat grains rely primarily on photosynthates from the flag leaves; thus, maintaining healthy leaves with high photosynthetic efficiency is critical for achieving high yields. However, in the North China Plain, winter wheat is highly vulnerable to seasonal dry-hot winds and drought during the grain-filling period, which is a critical stage in wheat cultivation [1]. Climate change has further exacerbated this issue [2, 3]. Dry-hot winds rapidly increase leaf transpiration while decreasing photosynthetic rates. Additionally, high temperatures impair the transport of assimilates and the degradation of primary proteins in plants, leading to insufficient grain filling and shriveled kernels. This dual stress severely compromises winter wheat yields [4]. Therefore, understanding the underlying biomolecular mechanisms to prevent premature senescence during the grain-filling stage is crucial for ensuring both crop yield and quality.

In 2022, the Ministry of Agriculture and Rural Affairs of China released the “Action Plan for Fertilizer Use Reduction by 2025,” aiming to decrease chemical fertilizer application without compromising crop yields, while simultaneously promoting the use of organic fertilizers. Proper fertilization is central to wheat cultivation, as the crop requires adequate nutrients to achieve optimal yields. The application of organic fertilizers can enhance chlorophyll content and photosynthetic efficiency, thereby improving winter wheat yields [5–7]. Organic fertilizers, such as livestock manure, directly increase soil available nutrients, promote microbial proliferation, and enhance soil water and nutrient retention capacities. Furthermore, they significantly alleviate problems such as soil acidification and nutrient leaching caused by the excessive use of chemical fertilizers [8]. However, some farmers have found that completely replacing chemical fertilizers with organic alternatives fails to meet crop demands and leads to yield reductions. To address this, organic-inorganic compound fertilizers have been developed to combine the benefits of both organic and inorganic sources. These are typically prepared by adding inorganic fertilizers to microbially fermented organic materials (e.g., animal manure, peat). Common examples include biochar-based fertilizers, humic acid fertilizers, and compost-enriched fertilizers. Their application differs from the separate use of organic and inorganic fertilizers in several ways: compound fertilizers offer convenience and a balanced nutrient supply, whereas the simultaneous but separate application of organic and inorganic fertilizers allows for greater flexibility and targeted nutrient management. The choice between these methods depends on factors such as crop requirements, soil conditions, resource availability, and farming practices.

Genetic studies have revolutionized wheat cultivation by enhancing nutrient use efficiency, improving root architecture, and increasing nutritional quality. For instance, genetic variations related to photosynthetic capacity have been identified among different wheat genotypes. These variations are linked to traits such as CO₂ assimilation rates and chlorophyll content, which directly impact photosynthetic efficiency [9]. Researchers have also identified wheat varieties with enhanced root systems that are more efficient at nutrient uptake. Through transcriptome analysis, specific genes expressed in high-quality wheat varieties have been characterized [10], and changes in transcription factor expression under drought stress have been elucidated [11]. Furthermore, variations in genes associated with chlorophyll stability and heat tolerance have been explored to develop heat-resistant wheat cultivars [12]. Other studies have identified key loci and genes influencing grain-filling capacity [13], leading to the breeding of cultivars with optimized grain-filling characteristics [14].

In this study, we investigated the effects of organic-inorganic compound fertilizers on the photosynthetic characteristics and yield of winter wheat. From a holistic “source-flow-sink” perspective, we examined the relationship between photosynthate supply in wheat leaves, the transport of non-structural carbohydrates (NSCs) in stem sheaths, and starch accumulation in grains. Furthermore, we employed high-throughput sequencing to analyze the impact of these fertilizers on gene expression at the transcriptomic level. These findings provide a theoretical basis for optimizing fertilization strategies in wheat cultivation to enhance both crop yield and quality.

## Materials and methods

### Materials and design

Field experiments were conducted from 2022 to 2025 at the experimental site of the Dezhou Academy of Agricultural Sciences, Dezhou, Shandong, China (115°45’ E, 36°43’ N). The winter wheat cultivar ‘Jimai 22’ was provided by the Shandong Academy of Agricultural Sciences. The soil at the site is classified as a calcareous fluvisol with a sandy loam texture. The region features a continental monsoon climate, with annual average sunshine hours, temperature, and precipitation of 2,724.8 h, 12.9 °C, and 439.5–593.5 mm, respectively. The initial soil properties (measured in 2021) were as follows: total nitrogen (TN), 1.24 g/kg; total phosphorus (TP), 0.83 g/kg; total potassium (TK), 20.80 g/kg; available nitrogen (AN), 58.12 mg/kg; available phosphorus (AP), 37.40 mg/kg; available potassium (AK), 282.00 mg/kg; soil organic matter (SOM), 20.39 g/kg; pH, 8.34; and soil salinity, 18 mg/kg.

Three fertilization treatments were evaluated: (1) chemical fertilizer alone (CK); (2) chemical fertilizer supplemented with composted pig manure (T1); and (3) organic-inorganic compound fertilizer (T2). Both the composted pig manure and the organic-inorganic compound fertilizer were purchased from Jinan Old Buddy Biological Fertilizer Co., Ltd. (Jinan, China). The chemical fertilizers, including urea, double superphosphate, and potassium sulfate, were obtained from Shandong Lianong Agricultural Materials Service Co., Ltd. (Jinan, China).

Each treatment was arranged in three replicate plots (5 m × 8 m each), with a 5-m buffer zone between adjacent plots. Within each plot, wheat was sown in rows spaced 1 m apart. Fertilizer application rates were consistent with typical local agricultural practices (Supplementary Tables 1–3). Fertilizers were broadcast on the soil surface prior to sowing, followed by mechanical tillage. Irrigation timing, frequency, and volume were kept uniform across all treatments, and all other field management practices adhered to local farming conventions. Samples for yield determination, physiological indicator analysis, and transcriptome analysis were all collected during the third year of the experiment.

### Yield and biomass determination

Wheat was harvested in June. Grain yield components were determined by measuring the number of spikes, the number of grains per spike, and the thousand-kernel weight (TKW) from the central area (1 m²) of each plot.

### Determination of physiological indexes of photosynthesis

Thirty flag leaves were randomly sampled from the central area (1 m²) of each plot during the grain-filling stage in mid-May. An LI-6800 portable photosynthesis system (LI-COR, Lincoln, NE, USA) was used to measure the following parameters of the flag leaves: net photosynthetic rate (Pn), stomatal conductance (Gs), intercellular CO₂ concentration (Ci), and transpiration rate (Tr). Chlorophyll content was determined via ethanol extraction. The activities of ribulose-1,5-bisphosphate carboxylase/oxygenase (Rubisco) and phosphoenolpyruvate carboxylase (PEPC) were assayed using a microplate reader.

### Analysis of carbohydrate transport characteristics and carbon metabolism-related enzymes activity

The contents of soluble sugars and starch were determined using the anthrone colorimetric method, while sucrose content was measured via the resorcinol colorimetric method. The activities of sucrose phosphate synthase (SPS), ADP-glucose pyrophosphorylase (AGPase), granule-bound starch synthase (GBSS), soluble starch synthase (SSS), and starch branching enzyme (SBE) were assayed using commercial kits purchased from Suzhou Keming Biotechnology Co., Ltd. (Suzhou, China).

$$\mathrm{NSC}= \mathrm{soluble}\:\mathrm{sugar}\:\mathrm{content}\:+\:\mathrm{starch}\:\mathrm{content}$$

$$\mathrm{NSC}\:\mathrm{accumulation}= \mathrm{NSC}\:\mathrm{content}\times\:\mathrm{dry}\:\mathrm{matter}\:\mathrm{weight}$$

$$\begin{aligned}\mathrm{NSC}\:\mathrm{transfer}\:\mathrm{rate}\:\mathrm{of}\:\mathrm{stem}\:\mathrm{sheath}&=\left(\mathrm{NSC}\:\mathrm{accumulation}\:\mathrm{at}\:\mathrm{flowering}\:\mathrm{stage}\:-\:\mathrm{NSC}\:\mathrm{accumulation}\:\mathrm{at}\:\mathrm{mature}\:\mathrm{stage}\right)\\&/\:\mathrm{NSC}\:\mathrm{accumulation}\:\mathrm{at}\:\mathrm{flowering}\:\mathrm{stage}\times100\%\end{aligned}$$

### Transcriptome sequencing and data analysis

Total RNA was extracted from flag leaves using a Plant RNA Extraction Kit (TaKaRa, Dalian, China). RNA quality and purity were assessed using an Agilent 2100 Bioanalyzer with an Agilent RNA 6000 Nano Kit (Agilent, Santa Clara, CA, USA). Messenger RNA (mRNA) was isolated via oligo(dT) enrichment and chemical fragmentation. Subsequently, cDNA libraries were constructed using a PrimeScript RT Reagent Kit (TaKaRa, Dalian, China) and sequenced on an Illumina NovaSeq 6000 platform to generate paired-end reads.

Raw image data were converted to FASTQ format using Casava. Low-quality reads were filtered out using Trimmomatic (v0.36) with the parameters “SLIDINGWINDOW:4:15 MINLEN:75”. High-quality clean reads were then aligned to the reference genome using Hisat2 (v2.0.5) in orientation mode. Gene expression levels were quantified using HTSeq, and differential expression analysis was performed using the R package edgeR. Differentially expressed genes (DEGs) were identified based on the criteria of |log₂FC|≥1 and a false discovery rate (FDR) or P-value ≤ 0.05.

Functional annotation of DEGs was performed via Gene Ontology (GO) enrichment and KEGG pathway analysis using Goatools and KOBAS, respectively. GO terms and metabolic pathways were considered significantly enriched if the Bonferroni-corrected P-value was less than 0.05. Transcription factors (TFs) were predicted by querying all genes against the PlantTFDB database with an E-value cutoff of ≤1e⁻⁵.

Data analysis was conducted on the Majorbio Cloud Platform (https://www.majorbio.com/), and gene expression patterns were visualized using TBtools. For weighted gene co-expression network analysis (WGCNA), genes with an FPKM value > 5 in at least 50% of the samples were selected. To minimize noise, genes were further screened based on median absolute deviation (MAD). WGCNA was performed using a soft threshold power of 26, a minimum module size of 60, and a combined module threshold of 0.30. Genes within identified modules were classified according to KEGG annotations.

### Real-time quantitative PCR (qRT-PCR)

Gene-specific primers were designed using Primer3 (https://bioinfo.ut.ee/primer3/) for the following target genes: TraesCS2B02G078900, TraesCS2B02G079200, TraesCS2D02G065300, TraesCS4A02G059800, TraesCS7A02G318700, and TraesCS2D02G065400. cDNA synthesis was performed following the protocol described in Sect.  2.4. qRT-PCR amplification was carried out under the following conditions: initial denaturation at 95 °C for 5 min, followed by 40 cycles of 95 °C for 30 s and 60 °C for 30 s. Gene expression levels were calculated using the 2^−ΔΔCT^ method, with the wheat actin gene serving as the internal reference. All experiments were performed in triplicate.

### Statistical analysis

All data were analyzed using SPSS 26.0 (IBM Corp., Armonk, NY, USA) and are presented as mean ± standard deviation (SD). Differences between treatment groups were evaluated using one-way analysis of variance (ANOVA), and statistical significance was defined as *P* < 0.05.

## Results

### Wheat yield

As shown in Table 1, wheat yield, biomass, and spike number all followed the order of T2 > T1 > CK, with all differences being statistically significant. Additionally, the thousand-kernel weight (TKW) in T2 was significantly higher than that in T1 and CK. However, no significant differences were observed in the number of grains per spike among the three treatments.

**Table 1:** Key parameters of wheat harvest

Treatment	Spike number (pieces·m^− 2^)	Grain number per spike	Thousand kernel weight (g)	Yield (kg·hm^− 2^)	Wheat biomass (kg·hm^− 2^)
CK	562.87 ± 35.93^c^	29.46 ± 1.10^a^	47.15 ± 1.01^b^	9468.85 ± 221.76^c^	16667.20 ± 327.89^c^
T1	650.70 ± 49.86^b^	29.73 ± 0.99^a^	46.97 ± 1.16^b^	9878.25 ± 89.95^b^	18173.55 ± 643.27^b^
T2	748.50 ± 11.98^a^	29.27 ± 0.95^a^	49.40 ± 0.75^a^	10527.75 ± 236.70^a^	19763.10 ± 579.06^a^

Note: Letters in superscript indicate significant differences between the treatments (*P *< 0.05)

### Photosynthesis

**Fig. 1:**
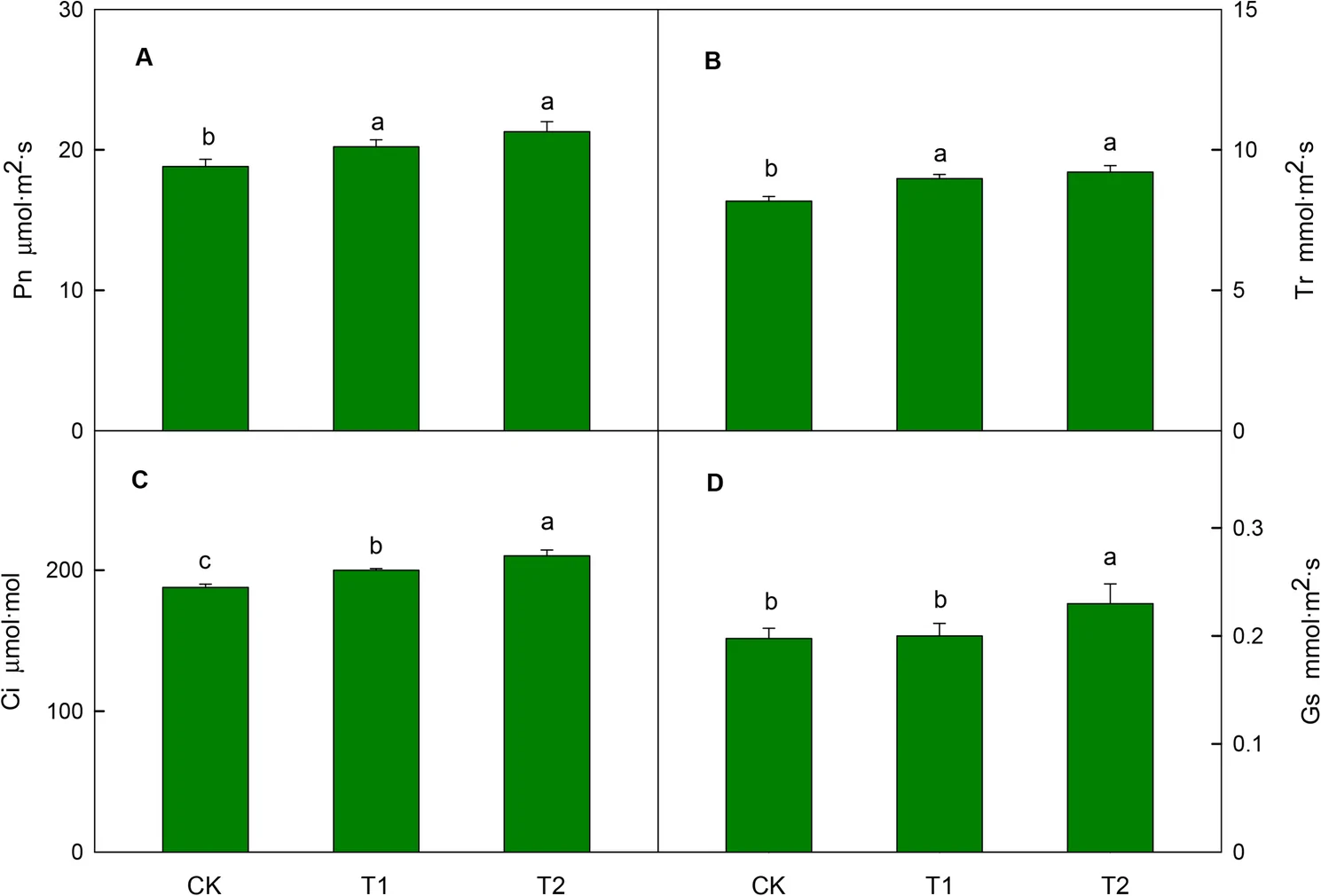
Photosynthetic characteristics of flag leaves

Compared to the CK treatment, both T1 and T2 exhibited significantly higher total chlorophyll and chlorophyll a contents in their flag leaves (Table 2), as well as higher net Pn and Tr (Fig. 1); however, no significant differences were observed between T1 and T2 for these parameters. Additionally, T2 showed significantly higher intercellular Ci and Gs than both T1 and CK (Fig. 1). These results indicate that the application of organic-inorganic compound fertilizer (T2) effectively enhanced the photosynthetic capacity of wheat.

**Table 2:** Chlorophyll content of the flag leaves (mg·g^− 1^)

Treatment	Total chlorophyll	Chlorophyll a	Chlorophyll b
CK	0.70 ± 0.05^b^	0.47 ± 0.03^b^	0.23 ± 0.03^a^
T1	0.77 ± 0.01^a^	0.52 ± 0.03^a^	0.25 ± 0.02^a^
T2	0.80 ± 0.06^a^	0.55 ± 0.03^a^	0.25 ± 0.02^a^

Note: Letters in superscript indicate significant differences between the treatments (*P* < 0.05)

**Fig. 2:**
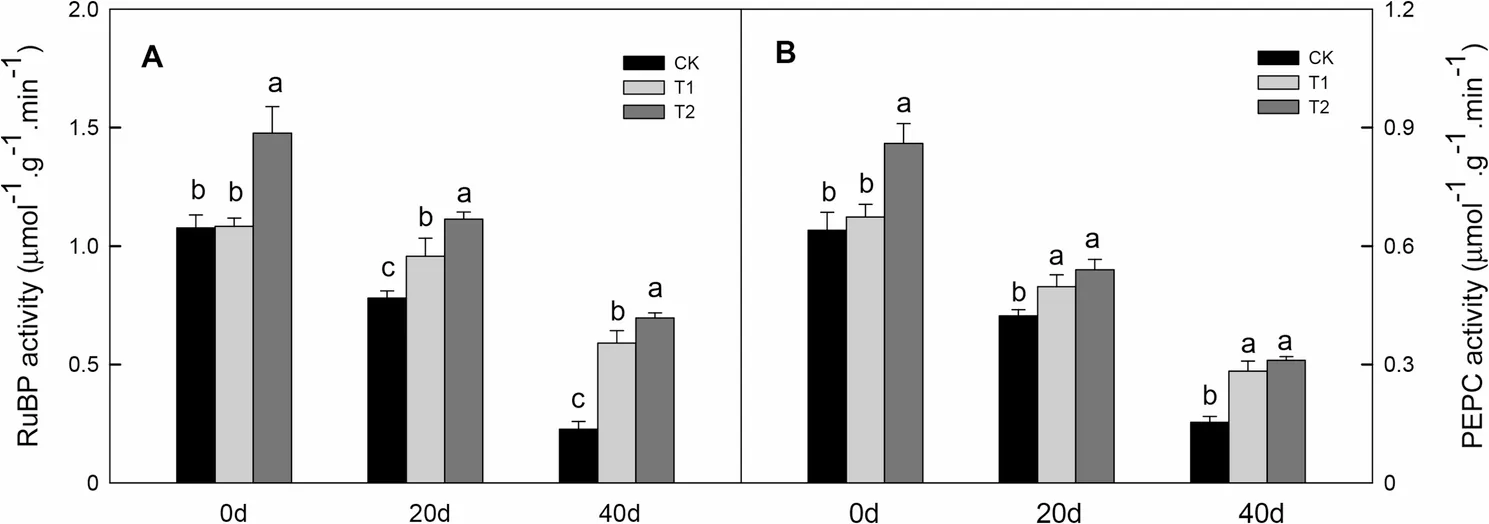
Enzyme activities related to photosynthesis in flag leaves

The activities of Rubisco and PEPC gradually decreased from the flowering stage to the maturity stage. Among the treatments, the T2 treatment consistently exhibited the highest enzyme activities. During the grain-filling stage, Rubisco activity in T2 was 42.31% and 15.63% higher than that in CK and T1, respectively. Furthermore, PEPC activity in T2 was significantly increased by 28.57% compared to CK, with no significant difference observed between T2 and T1 (Fig. 2).

### Carbohydrate transport and accumulation

#### NSC transport rate and accumulation

The transport rate of non-structural carbohydrates (NSCs) in the stem sheaths and the accumulation of NSCs in the grains were significantly higher in the T2 treatment than in the CK and T1 treatments. Specifically, the NSC transport rate in the stem sheaths of T2 increased significantly by 23.84% and 20.38% compared to CK and T1, respectively. Similarly, NSC accumulation in the grains increased significantly by 15.80% and 18.94%, respectively. No significant differences were observed between the CK and T1 treatments (Table 3).

**Table 3:** Characteristics of carbohydrate accumulation and transport

	Stem sheath NSC content (mg·g^− 1^)	Stem sheath NSC transport rate (%)	Grain NSC content (mg·g^− 1^)	Accumulation of grain NSC (kg·hm^− 2^)
CK	196.38 ± 4.39^a^	22.99 ± 0.95^b^	702.81 ± 26.32^b^	6943.98 ± 316.58^b^
T1	195.29 ± 3.17^a^	23.65 ± 2.20^b^	713.69 ± 28.04^b^	6761.17 ± 402.52^b^
T2	195.21 ± 1.64^a^	28.47 ± 2.04^a^	763.88 ± 14.27^a^	8041.45 ± 208.66^a^

Note: Letters in superscript indicate significant differences between the treatments (*P *< 0.05)

#### Enzyme activities related to carbohydrate accumulation and transport

**Fig. 3:**
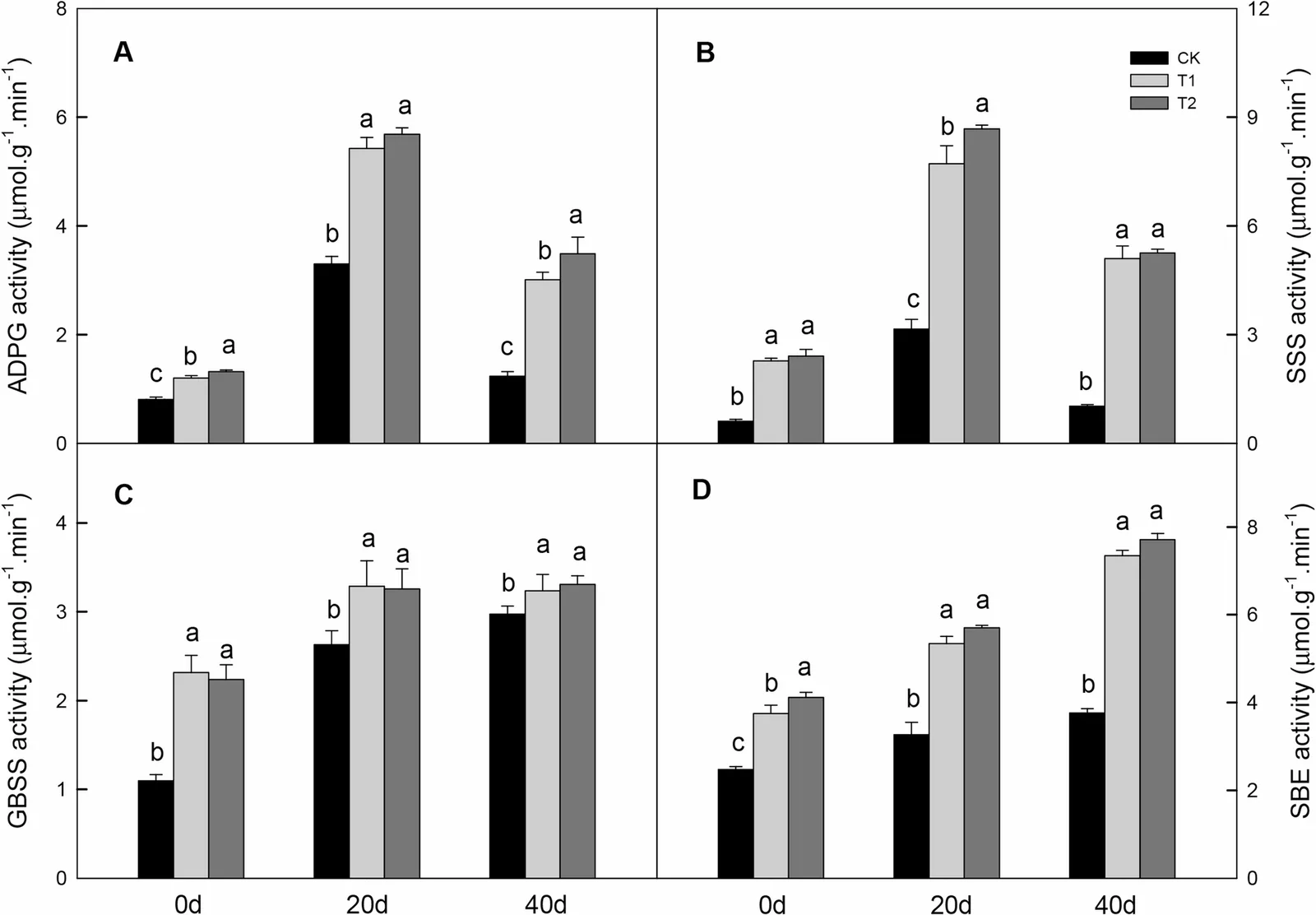
Enzyme activities related to carbon metabolism in wheat grains

The activities of ADPG and SSS exhibited a trend of initially increasing and then decreasing from the flowering stage to the maturity stage. Among the treatments, ADPG activity in T2 during the grain-filling stage was significantly higher than that in CK by 72.4%, with no significant difference observed between T2 and T1. Furthermore, SSS activity in T2 was 175.24% and 12.45% higher than that in the CK and T1 treatments during the grain-filling stage, respectively (Fig. 3a and b).

In contrast, GBSS and SBE activities increased gradually, reaching their peak levels at the maturity stage. During the grain-filling stage, the activities of GBSS and SBE in the grains of T2 were significantly higher than those in CK by 23.95% and 74.62%, respectively, with no significant differences observed between T2 and T1.

### Sequencing data statistics

A total of 650,148,242 clean reads were obtained from the RNA-seq data of the CK, T1, and T2 treatments. The nine samples in this study exhibited a mean Q30 value of 94.42% and a mean Q20 value of 98.02%, indicating high-quality sequencing data. Principal component analysis (PCA) based on FPKM values successfully separated the CK, T1, and T2 groups, demonstrating the strong discriminatory power of the transcriptomic data (Supplementary Fig. 1). Additionally, hierarchical clustering revealed that the T1 and T2 samples exhibited distinct differentially expressed gene (DEG) expression patterns compared to the CK samples (Supplementary Fig. 2).

### Differential gene expression

#### GO and KEGG analysis of DEGs

**Fig. 4:**
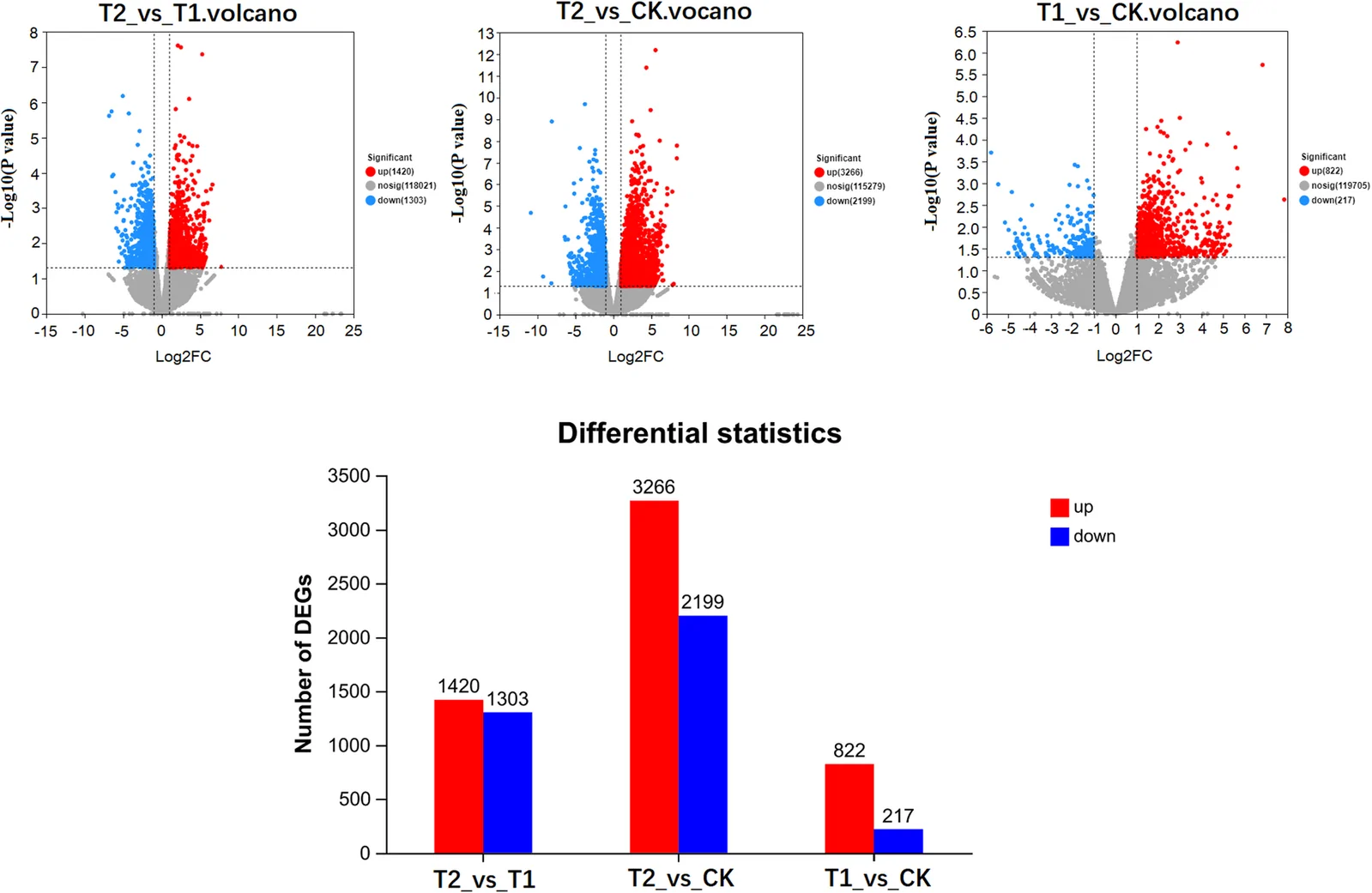
Differential expression of genes in flag leaves

Figure 4 illustrates the number of differentially expressed genes (DEGs) among the treatments, while Table 4 summarizes their functional annotations. In the T2 vs. T1 comparison, the 1,420 up-regulated genes were primarily enriched in photosynthesis and the carbon cycle, whereas the 1,303 down-regulated genes were mainly associated with oxidation-reduction processes. In the T2 vs. CK comparison, the 3,266 up-regulated genes were predominantly involved in photosynthesis and cellular responses to light stimulus, while the 2,199 down-regulated genes were mainly linked to chloride ion transmembrane transport and chaperone-mediated protein folding. Finally, in the T1 vs. CK comparison, the 822 up-regulated genes were primarily associated with glycosyltransferase activity, and the 217 down-regulated genes were mainly involved in the carbon cycle and photosynthesis.

**Table 4:** GO enrichment analysis (top 10) of differentially expressed genes

Comparison	Up-regulation	Down-regulation
T2 vs. T1	ribulose-bisphosphate carboxylase activity	regulation of reactive oxygen species biosynthetic process
	carbon fixation	pheophorbide a oxygenase activity
	photosynthesis, dark reaction	dehydrodolichyl diphosphate synthase complex
	reductive pentose-phosphate cycle	ferredoxin hydrogenase activity
	photorespiration	oxidoreductase activity, acting on hydrogen as donor, iron-sulfur protein as accept
	cellular metabolic compound salvage	oxidoreductase activity, acting on hydrogen as donor
	carboxy-lyase activity	periplasmic space
	carbon-carbon lyase activity	signal peptidase complex
	Photosynthesis	oxidoreductase activity, acting on iron-sulfur proteins as donors
	photosynthetic membrane	endoplasmic reticulum protein-containing complex
T2 vs. CK	ribulose-bisphosphate carboxylase activity	chloride transmembrane transporter activity
	photosynthesis, dark reaction	chaperone binding
	reductive pentose-phosphate cycle	ATP-dependent protein folding chaperone
	photorespiration	organelle lumen
	cellular response to light stimulus	intracellular organelle lumen
	response to light stimulus	membrane-enclosed lumen
	response to radiation	protein folding
	UDP-glycosyltransferase activity	endoplasmic reticulum
	DNA-binding transcription factor activity	bounding membrane of organelle
	transcription regulator activity	nitrogen compound transport
T1 vs. CK	UDP-glycosyltransferase activity	ribulose-bisphosphate carboxylase activity
	Hexosyl transferase activity	reductive pentose-phosphate cycle
	DNA-binding transcription factor activity	photosynthesis, dark reaction
	Glycosyl transferase activity	photorespiration
	transcription regulator activity	carbon fixation
	regulation of DNA-templated transcription	sucrose alpha-glucosidase activity
	regulation of RNA biosynthetic process	alpha-glucosidase activity
	regulation of nucleic acid-templated transcription	beta-fructofuranosidase activity
	regulation of RNA metabolic process	cellular metabolic compound salvage
	regulation of nucleobase-containing compound	alpha-amino acid catabolic process

Table 5 presents the results of the KEGG pathway enrichment analysis. In the T2 vs. T1 comparison, the top three enriched pathways for up-regulated genes were glyoxylate and dicarboxylate metabolism, carbon fixation in photosynthetic organisms, and photosynthesis - antenna proteins. Conversely, the top three pathways for down-regulated genes were protein processing in the endoplasmic reticulum, protein export, and valine, leucine, and isoleucine degradation. For T2 vs. CK, the most significantly enriched pathways among up-regulated genes were plant circadian rhythm, carbon fixation in photosynthetic organisms, and glyoxylate and dicarboxylate metabolism, whereas the top three for down-regulated genes were protein processing in the endoplasmic reticulum, valine, leucine, and isoleucine degradation, and various types of N-glycan biosynthesis. Finally, in the T1 vs. CK comparison, the top three up-regulated pathways were flavone and flavonol biosynthesis, plant hormone signal transduction, and glutathione metabolism. The top three down-regulated pathways in this comparison were carbon fixation in photosynthetic organisms, glyoxylate and dicarboxylate metabolism, and ascorbate and aldarate metabolism.

**Table 5:** KEGG enrichment analysis (top 10) of differentially expressed genes

Comparison	Upregulation	Downregulation
T2 vs. T1	Glyoxylate and dicarboxylate metabolism	Protein processing in endoplasmic reticulum
	Carbon fixation in photosynthetic organisms	Protein export
	Photosynthesis - antenna proteins	Valine, leucine and isoleucine degradation
	Circadian rhythm - plant	MAPK signaling pathway - plant
	Carotenoid biosynthesis	Plant-pathogen interaction
	Nitrogen metabolism	Glucosinolate biosynthesis
	Flavonoid biosynthesis	Valine, leucine and isoleucine biosynthesis
	Porphyrin metabolism	Benzoxazinoid biosynthesis
	Taurine and hypotaurine metabolism	Fatty acid degradation
	Glycine, serine and threonine metabolism	beta-Alanine metabolism
T2 vs. CK	Circadian rhythm - plant	Protein processing in endoplasmic reticulum
	Carbon fixation in photosynthetic organisms	Valine, leucine and isoleucine degradation
	Glyoxylate and dicarboxylate metabolism	Various types of N-glycan biosynthesis
	Nitrogen metabolism	N-Glycan biosynthesis
	Alanine, aspartate and glutamate metabolism	Pyruvate metabolism
	Taurine and hypotaurine metabolism	Valine, leucine and isoleucine biosynthesis
	Photosynthesis - antenna proteins	Ubiquinone and other terpenoid-quinone biosynthesis
	Flavone and flavonol biosynthesis	beta-Alanine metabolism
	Starch and sucrose metabolism	Fatty acid degradation
	Cyanoamino acid metabolism	Folate biosynthesis
T1 vs. CK	Flavone and flavonol biosynthesis	Carbon fixation in photosynthetic organisms
	Plant hormone signal transduction	Glyoxylate and dicarboxylate metabolism
	Glutathione metabolism	Ascorbate and aldarate metabolism
	Starch and sucrose metabolism	Glycine, serine and threonine metabolism
	Tryptophan metabolism	Glutathione metabolism
	Nitrogen metabolism	Phenylpropanoid biosynthesis
	Isoflavonoid biosynthesis	DNA replication
	Cutin, suberine and wax biosynthesis	Phenylalanine, tyrosine and tryptophan biosynthesis
	Flavonoid biosynthesis	Biosynthesis of various plant secondary metabolism
	MAPK signaling pathway – plant	Phenylalanine metabolism

**Fig. 5:**
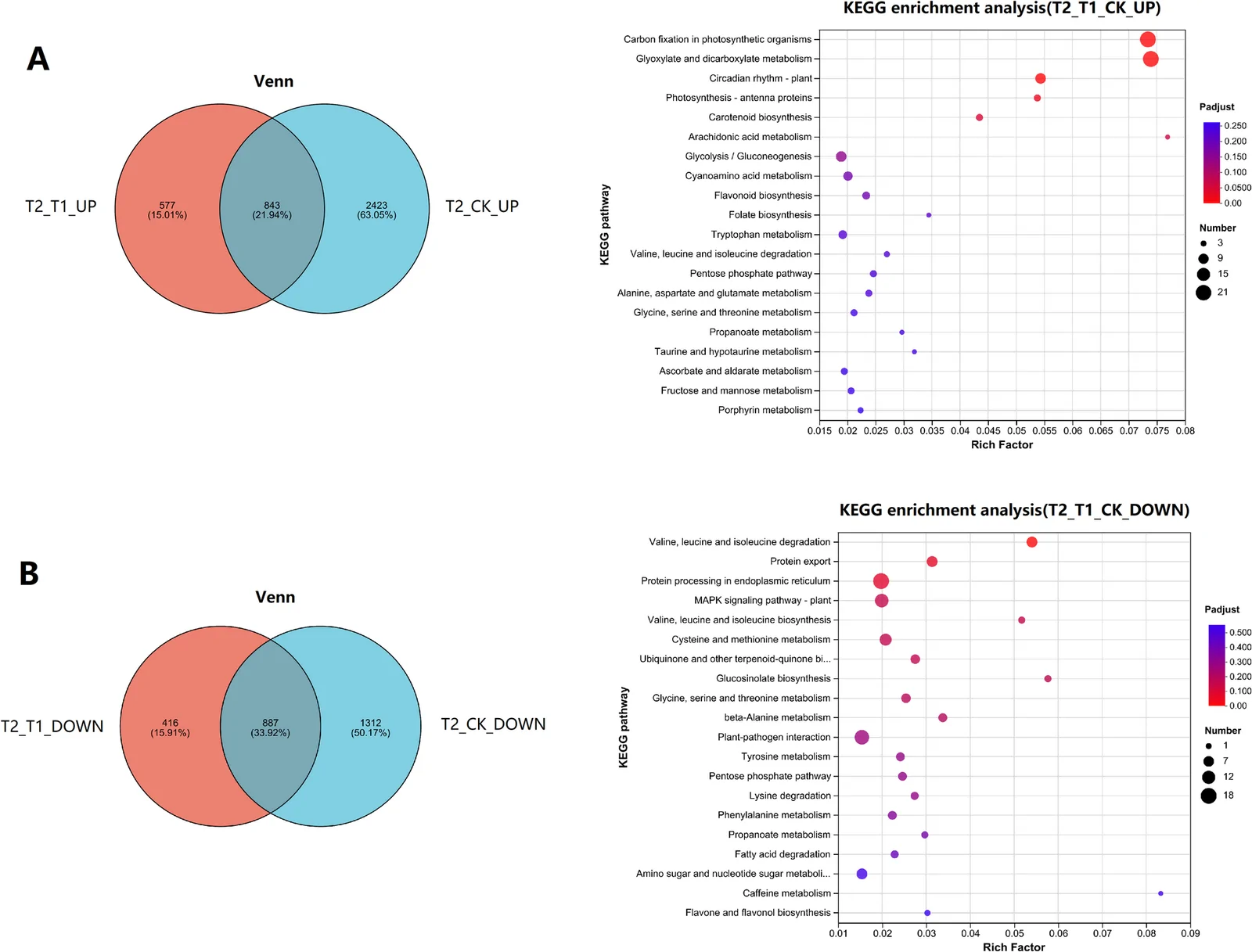
KEGG pathways of differentially expressed genes in the T2 treatment

By analyzing the intersection of the T2 vs. T1 and T2 vs. CK comparisons, 843 commonly up-regulated genes were identified. The top enriched KEGG pathways for these genes included glyoxylate and dicarboxylate metabolism, carbon fixation in photosynthetic organisms, and plant circadian rhythm (Fig. 5)

#### Transcription factor (TF) analysis of DEGs

**Fig. 6:**
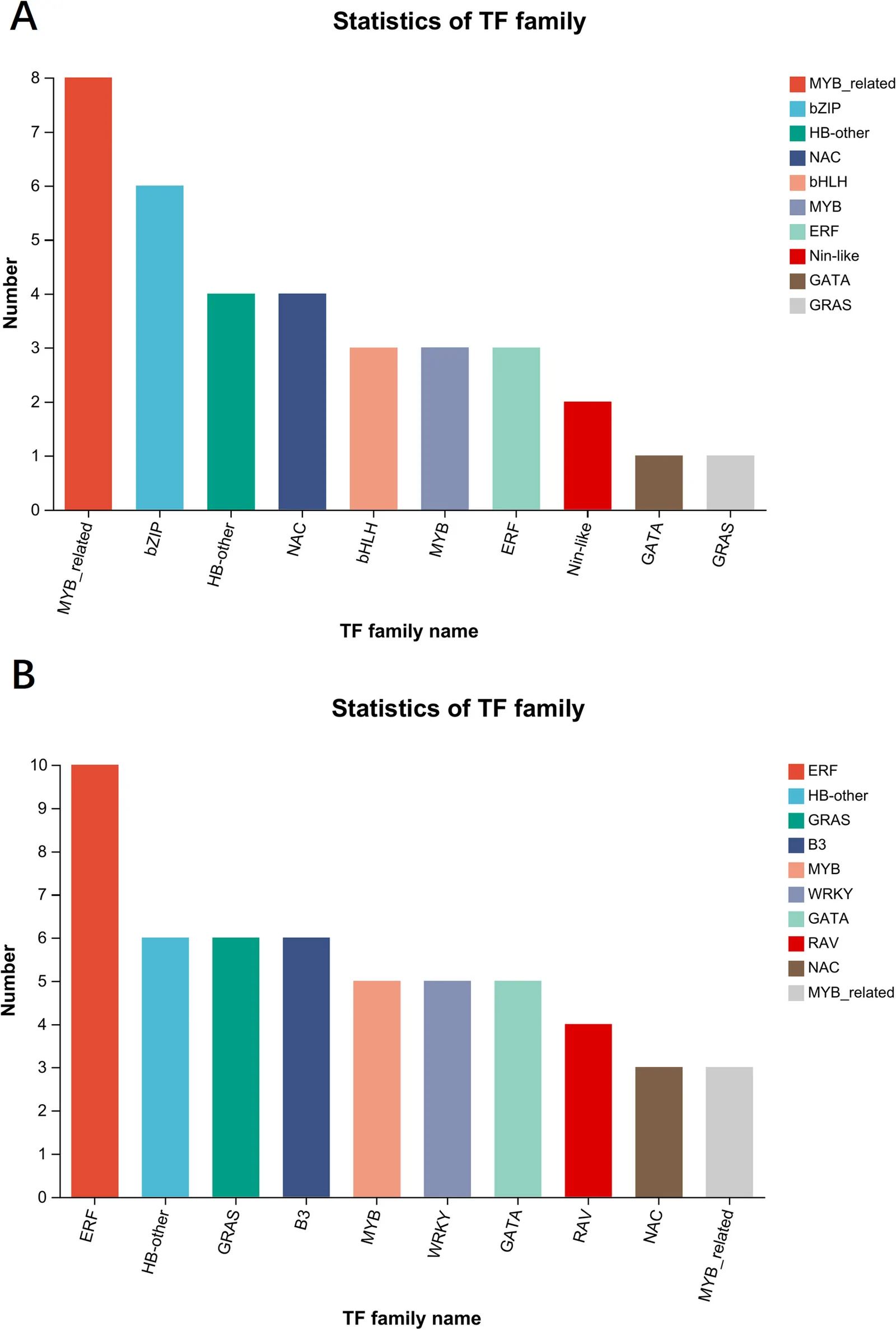
Transcription factors of differentially expressed genes in the T2 treatment

In this study, a total of 385 transcription factor (TF) genes belonging to 34 TF families were identified. The TF families most frequently represented among the differentially expressed genes (DEGs) in T2 were MYB-related, bZIP, ERF, and HB-other. Among the 68 up-regulated DEGs, the encoded TFs primarily belonged to the MYB-related, bZIP, and HB-other families. Conversely, the 108 down-regulated DEGs were mainly classified into the ERF, HB-other, and GRAS families (Fig. 6; Table 6).

**Table 6:** Differentially expressed genes in the T2 treatment and their transcription factors

	Gene ID	Gene description	Family
UP	TraesCS1B02G449100	Myb-like DNA-binding domain	MYB_related
	TraesCS2D02G437600	Myb-like DNA-binding domain	MYB_related
	TraesCS3A02G216200	Myb-like DNA-binding domain	MYB_related
	TraesCS3A02G361800	Myb-like DNA-binding domain	MYB_related
	TraesCS3D02G217800	Myb-like DNA-binding domain	MYB_related
	TraesCS7A02G146700	Myb-like DNA-binding domain	MYB_related
	TraesCS7B02G251400	Myb-like DNA-binding domain	MYB_related
	TraesCS7D02G347500	Myb-like DNA-binding domain	MYB_related
	TraesCS5B02G059200	bZIP transcription factor	bZIP
	TraesCS6D02G167800	bZIP transcription factor	bZIP
	TraesCS1A02G311900	bZIP transcription factor	bZIP
	TraesCS6A02G175800	bZIP transcription factor	bZIP
	TraesCS6B02G209600	bZIP transcription factor	bZIP
	TraesCS7A02G170600	bZIP transcription factor	bZIP
	TraesCS4D02G111900	Homeobox domain	HB-other
	TraesCS4D02G340700	Homeobox domain	HB-other
	TraesCS5A02G514700	Homeobox domain	HB-other
	TraesCS4D02G019600	Homeobox domain	HB-other
Down	TraesCS6B02G199900	AP2 domain	ERF
	TraesCS6D02G161300	AP2 domain	ERF
	TraesCS7A02G264100	AP2 domain	ERF
	TraesCS7B02G162100	AP2 domain	ERF
	TraesCS1D02G378500	AP2 domain	ERF
	TraesCS3B02G064900	AP2 domain	ERF
	TraesCS3B02G293000	AP2 domain	ERF
	TraesCS3D02G052300	AP2 domain	ERF
	TraesCS5B02G118200	AP2 domain	ERF
	TraesCS6B02G199800	AP2 domain	ERF
	TraesCS3B02G373800	Homeobox domain	HB-other
	TraesCS3D02G335500	Homeobox domain	HB-other
	TraesCS4D02G236600	Homeobox domain	HB-other
	TraesCS6B02G321100	Homeobox domain	HB-other
	TraesCS3A02G341700	Homeobox domain	HB-other
	TraesCS7A02G423800	Homeobox domain	HB-other
	TraesCS3A02G487500	GRAS domain family	GRAS
	TraesCS3B02G535100	GRAS domain family	GRAS
	TraesCS3D02G482600	GRAS domain family	GRAS
	TraesCS7A02G014200	GRAS domain family	GRAS
	TraesCS4D02G040400	GRAS domain family	GRAS
	TraesCS7D02G011400	GRAS domain family	GRAS

#### Genes related to photosynthesis

**Fig. 7:**
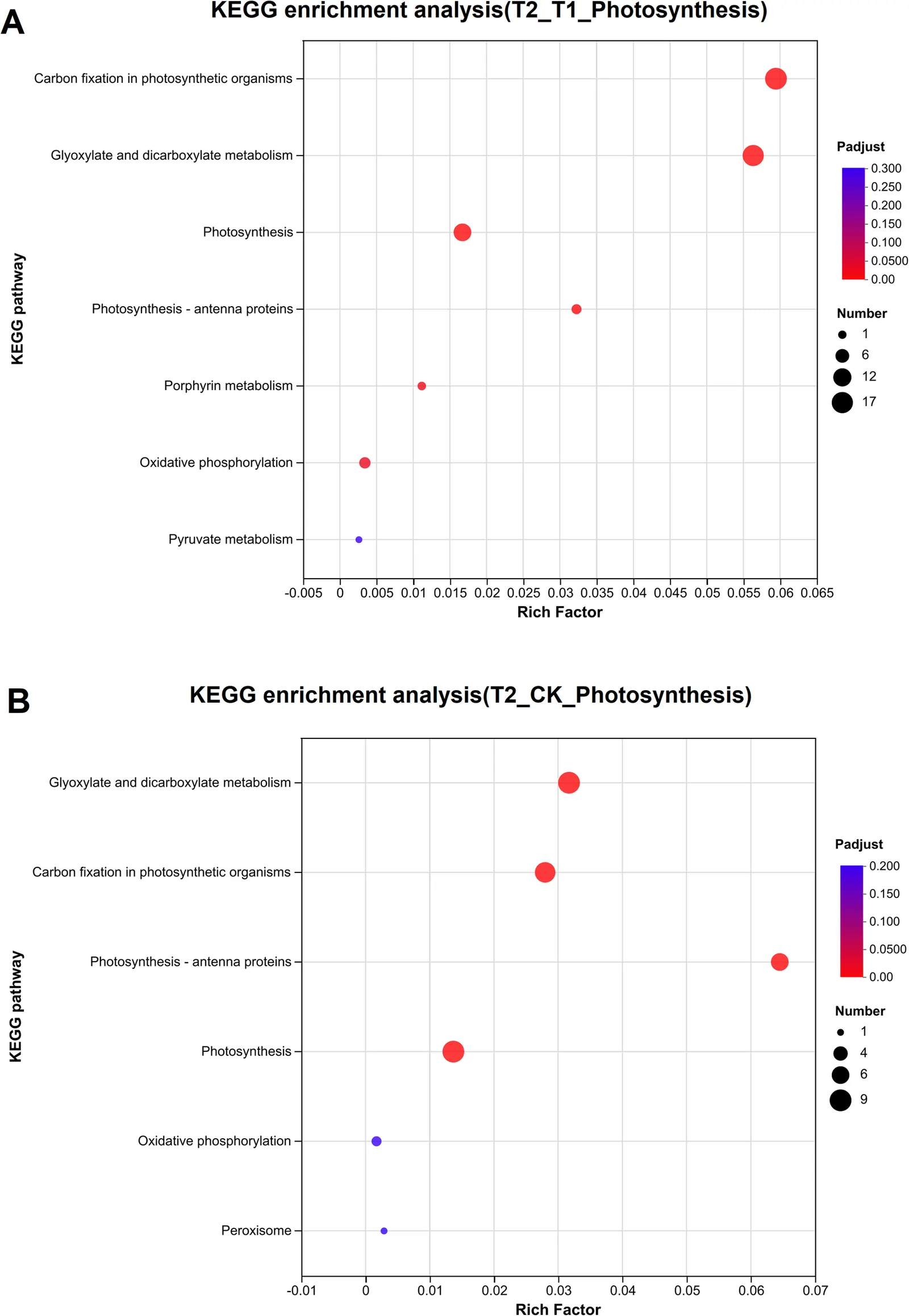
KEGG enrichment analysis of photosynthetic genes that were expressed differentially in T2 treatment

The differentially expressed genes (DEGs) involved in photosynthetic pathways were further analyzed (Tables 7 and 8). In the T2 vs. T1 comparison, the top three enriched KEGG pathways were carbon fixation in photosynthetic organisms, glyoxylate and dicarboxylate metabolism, and photosynthesis - antenna proteins (Fig. 7A). Conversely, for the T2 vs. CK comparison, the most significantly enriched pathways were photosynthesis - antenna proteins, glyoxylate and dicarboxylate metabolism, and carbon fixation in photosynthetic organisms (Fig. 7B).

**Table 7:** KEGG enrichment analysis of photosynthetic genes up-regulated in the T2 treatment compared to the CK treatment

Pathway ID	Description	Number of genes (*n* = 25)	*P*	*P*_adjust_	Genes
map00630	Glyoxylate and dicarboxylate metabolism	9	5.98E-14	3.59E-13	TraesCS2D02G065600 TraesCS2A02G250800 TraesCS2A02G067300 TraesCS2B02G079500 TraesCS5B02G162600 TraesCS5A02G165700 TraesCS5D02G169900 TraesCS5D02G169600 TraesCS2A02G067100
map00710	Carbon fixation in photosynthetic organisms	8	4.95E-12	1.48E-11	TraesCS2D02G065600 TraesCS2A02G067300 TraesCS2B02G079500 TraesCS5B02G162600 TraesCS5A02G165700 TraesCS5D02G169900 TraesCS5D02G169600 TraesCS2A02G067100
map00196	Photosynthesis - antenna proteins	6	1.97E-11	3.95E-11	TraesCS5D02G357600 TraesCS5D02G464800 TraesCS5B02G322900 TraesCS1A02G403300 TraesCS7D02G276300 TraesCS5A02G322500
map00195	Photosynthesis	9	1.08E-10	1.63E-10	TraesCS5B02G256400 TraesCS6B02G227900 TraesCS7D02G322100 TraesCS7B02G226200 TraesCS7A02G325500 TraesCS4D02G014200 TraesCS1A02G140100 TraesCS6A02G374400 TraesCS6D02G187800

**Table 8:** KEGG enrichment analysis of photosynthetic genes up-regulated in the T2 treatment compared to the T1 treatment

Pathway ID	Description	Number of genes (*n* = 37)	*P*	*P*_adjust_	Genes
map00710	Carbon fixation in photosynthetic organisms	17	1.89E-27	1.32E-26	TraesCS2D02G065600 TraesCS2D02G065200 TraesCS2B02G079500 TraesCS2A02G066700 TraesCS2B02G079100 TraesCS2D02G065100 TraesCS2A02G066800 TraesCS5D02G169900 TraesCS2D02G065400 TraesCS5D02G169600 TraesCS2D02G065300 TraesCS2A02G067100 TraesCS5B02G162600 TraesCS5A02G165700 TraesCS2A02G067200 TraesCS7D02G333900 TraesCS2B02G079300
map00630	Glyoxylate and dicarboxylate metabolism	16	2.06E-25	7.22E-25	TraesCS2D02G065600 TraesCS2D02G065200 TraesCS2B02G079500 TraesCS2A02G066700 TraesCS2B02G079100 TraesCS2D02G065100 TraesCS5A02G165700 TraesCS5D02G169900 TraesCS2D02G065400 TraesCS5D02G169600 TraesCS2D02G065300 TraesCS2A02G067100 TraesCS5B02G162600 TraesCS2A02G067200 TraesCS2A02G066800 TraesCS2B02G079300
map00195	Photosynthesis	11	9.78E-12	2.28E-11	TraesCS2D02G322700 TraesCS1B02G404700 TraesCS6D02G187800 TraesCS7A02G000400 TraesCS7B02G226200 TraesCS5A02G523400 TraesCS7A02G325500 TraesCS4D02G014200 TraesCS4A02G297900 TraesCS6B02G227900 TraesCS3D02G366600
map00196	Photosynthesis - antenna proteins	3	8.29E-05	0.000145	TraesCS5D02G219100 TraesCS7A02G276400 TraesCS7D02G276300
map00860	Porphyrin metabolism	2	0.011413	0.015978	TraesCS2A02G134000 TraesCS2D02G136200
map00190	Oxidative phosphorylation	4	0.020623	0.02406	TraesCS1B02G404700 TraesCS4A02G116500 TraesCS2A02G264700 TraesCS1D02G181300

#### qRT-PCR analysis of differentially expressed genes

To verify the accuracy of the transcriptome sequencing results, we selected six genes with differential expressions for verification by qRT-PCR. The qRT-PCR results agreed very well with the transcriptome sequencing data (Table 9). The quantitative comparison between the RNA-seq and qRT-PCR results demonstrated a high correlation between the two datasets (Supplementary Figures. 3).

**Table 9:** qRT-PCR analysis of selected genes

Gene ID	Gene Description	Function	Expression level		
			CK	T1	T2
TraesCS2B02G078900	Ribulose bisphosphate carboxylase small chain [Source: UniProtKB/TrEMBL; Acc: A0A341QH81]	Glyoxylate and dicarboxylate metabolism	5.22	3.96	3.03
TraesCS2B02G079200	Ribulose bisphosphate carboxylase small chain [Source: UniProtKB/TrEMBL; Acc: A0A341QH86]	Carbon fixation in photosynthetic organisms	7.39	5.63	5.55
TraesCS2D02G065300	Ribulose bisphosphate carboxylase small chain [Source: UniProtKB/TrEMBL; Acc: A0A1D5UUS0]	Glyoxylate and dicarboxylate metabolism	4.73	3.95	4.49
TraesCS4A02G059800	Branched-chain-amino-acid aminotransferase [Source: UniProtKB/TrEMBL; Acc: W5DXU1]	Valine, leucine and isoleucine degradation	3.06	4.45	4.44
TraesCS7A02G318700	Hexosyltransferase [Source: UniProtKB/TrEMBL; Acc: W5FEK1]	Protein processing in endoplasmic reticulum	2.35	0.94	3.07
TraesCS2D02G065400	Ribulose bisphosphate carboxylase small chain [Source: UniProtKB/TrEMBL; Acc: A0A1D5UUS1]	Carbohydrate metabolism	6.71	9.02	9.82

### Weighted gene co-expression network analysis

**Fig. 8:**
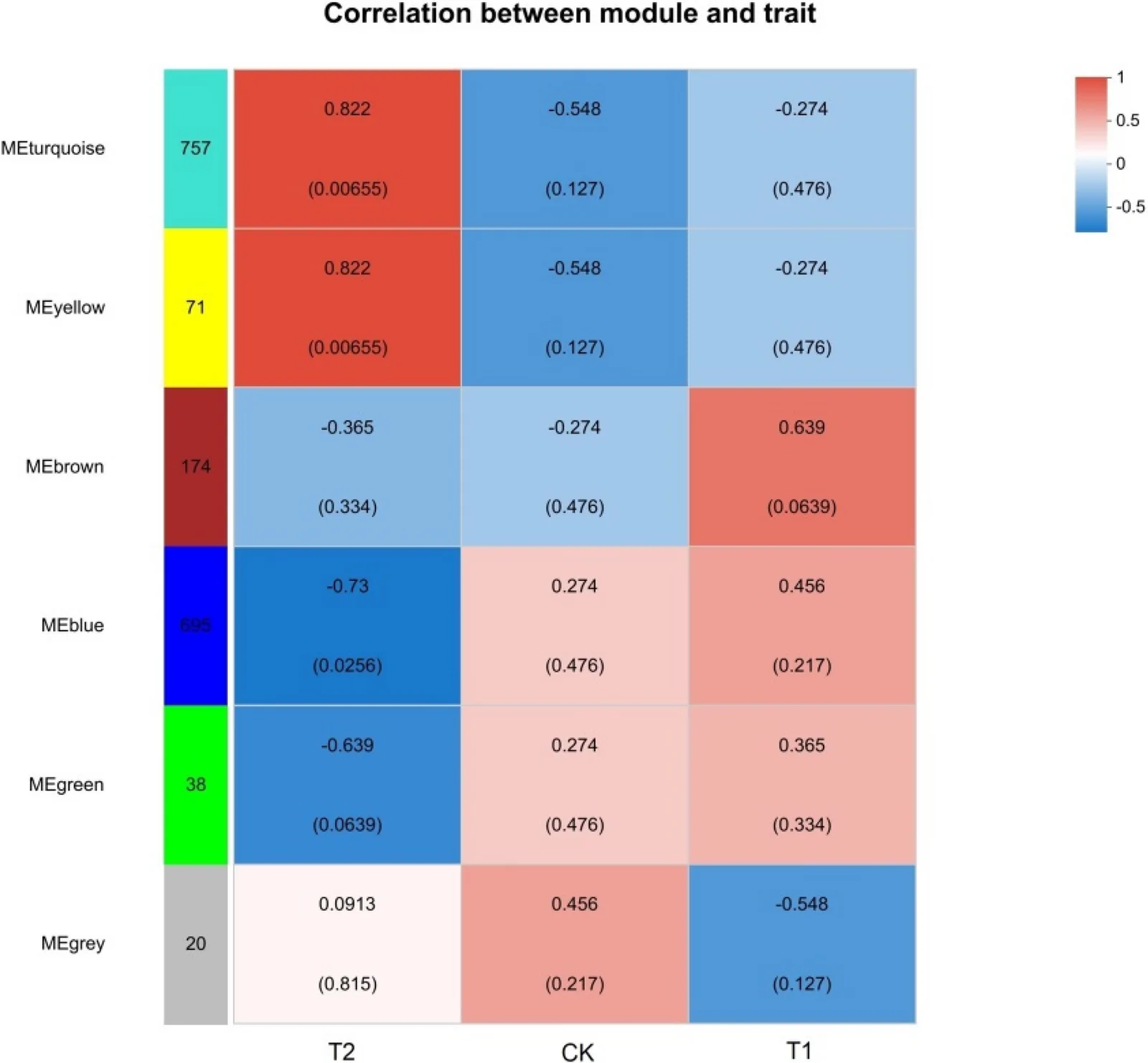
Weighted gene co-expression network analysis

WGCNA clustered the DEGs into six distinct modules (Fig. 8). Among these, two modules exhibited a significant positive correlation with the T2 treatment, while one module showed a significant negative correlation; no other significant correlations were observed. Table 10 summarizes the KEGG pathway enrichment analysis for the genes within each module. For the modules positively correlated with T2, the top enriched KEGG pathways were glyoxylate and dicarboxylate metabolism, carbon fixation in photosynthetic organisms, and photosynthesis - antenna proteins in the turquoise module, and beta-alanine metabolism and pantothenate and CoA biosynthesis in the yellow module. Conversely, the top enriched pathways for the module negatively correlated with T2 were protein processing in the endoplasmic reticulum, galactose metabolism, and ether lipid metabolism.

**Table 10:** KEGG pathways of genes significantly correlated with T2 (breakdown by cluster)

Module	KEGG ID	Pathway	Genes in module	*P* adjust
Turquoise	map00630	Glyoxylate and dicarboxylate metabolism	30	4.22E-22
	map00710	Carbon fixation in photosynthetic organisms	25	1.37E-16
	map00196	Photosynthesis - antenna proteins	8	3.25E-05
	map00195	Photosynthesis	17	0.000935
	map00906	Carotenoid biosynthesis	6	0.006988
	map00480	Glutathione metabolism	13	0.009861
	map00670	One carbon pool by folate	4	0.010349
Yellow	map00410	beta-Alanine metabolism	2	0.019948
	map00770	Pantothenate and CoA biosynthesis	2	0.031402
Blue	map04141	Protein processing in endoplasmic reticulum	21	6.91E-05
	map00052	Galactose metabolism	10	0.000198
	map00565	Ether lipid metabolism	5	0.012237
	map00590	Arachidonic acid metabolism	3	0.042103

## Discussion

Nutrient absorption and photosynthesis are key drivers of thousand-kernel weight (TKW), which is ultimately crucial for increasing winter wheat yield. As the final component of wheat yield, grain weight is directly proportional to overall yield. The starch required for grain weight accumulation is generally derived from two sources. One is the photosynthetic assimilates produced by the green vegetative organs after flowering. The other is the excess carbohydrates generated by photosynthetic organs before flowering, which are temporarily stored in organs such as the stem sheath and subsequently redistributed post-flowering [15, 16]. Substituting chemical fertilizers with organic fertilizers has been reported to increase both the chlorophyll content and the net photosynthetic rate in wheat [5, 6]. Consistent with this, our study found that the flag leaves in both the T2 and T1 treatments had significantly higher chlorophyll content, net photosynthetic rate, and transpiration rate compared to the control (CK). Stronger photosynthesis during the grain-filling stage provides the foundation for dry matter accumulation in wheat kernels, thereby enhancing yield. Furthermore, the rate of non-structural carbohydrate (NSC) transport in the stem sheath was significantly higher in the T2 treatment than in the other two treatments, providing the driving force for carbohydrate translocation. Ultimately, wheat grain weight is determined by the grain-filling process; the significantly higher starch accumulation observed in T2 grains compared to CK and T1 is a critical factor contributing to the increased grain weight.

At the transcriptomic level, the KEGG pathways enriched with up-regulated photosynthetic genes in T2 were primarily associated with glyoxylate and dicarboxylate metabolism, photosynthesis - antenna proteins, and carbon fixation in photosynthetic organisms. WGCNA further confirmed that these three pathways were significantly positively correlated with the T2 treatment. Glyoxylate and dicarboxylate metabolism is closely linked to photorespiration, helping to maintain the balance between energy absorption and metabolism while also contributing to plant stress resistance [17]. Antenna proteins are integral components of the light-harvesting complexes in photosynthetic organisms. They capture and transfer light energy to the reaction centers of photosystems I and II, and dissipate excess energy through non-photochemical quenching and other mechanisms to prevent photodamage. These proteins are essential for improving photosynthetic efficiency in agriculture [18, 19], and previous studies have examined how their phosphorylation and the formation of supercomplexes regulate photosynthetic efficiency [20, 21]. Through photosynthesis, plants assimilate CO2 to synthesize sugars and other organic compounds necessary for growth [22]. Carbon fixation specifically refers to the combination of one CO2 molecule with one C5 molecule to form two C3 molecules [23]. Additionally, WGCNA identified beta-alanine metabolism in the yellow module as being significantly positively correlated with the T2 treatment. A comprehensive review of the literature indicates that β-alanine, an abundant non-protein amino acid, serves not only as a precursor of coenzyme A but also plays crucial regulatory and protective roles in response to various environmental stresses, including hypoxia, waterlogging, drought, heat, and heavy metal stress [24, 25]. In T2, the up-regulation of photosynthesis-related genes fully demonstrates that the organic-inorganic compound fertilizer significantly alters the transcriptomic profile of wheat leaves during the grain-filling stage, suggesting its potential role in regulating both photosynthesis and grain fi.

## Conclusions

In this study, the physiological characteristics of wheat leaf photosynthesis, carbohydrate metabolism, and grain starch accumulation were investigated from a ‘source-flow-sink’ perspective following the application of organic-inorganic compound fertilizers. Among the three fertilization regimes typical of local agricultural practices, the T2 treatment produced the highest wheat yield, primarily driven by an increase in TKW. During the grain-filling stage, the T2 treatment exhibited the highest chlorophyll content, photosynthesis-related enzyme activities, and expression of photosynthesis-related genes in the flag leaves compared to both T1 and the control (CK). Therefore, the application of organic-inorganic compound fertilizers represents a feasible strategy for optimizing fertilizer management.

## Supplementary Information

Supplementary Material 1.

## Data Availability

The datasets generated during the current study are available in the NCBI database repository PRJNA1484397 (SRR39423835; SRR39423834; SRR39423833; SRR39423832; SRR39423831; SRR39423830; SRR39423829; SRR39423827; SRR39423828).
