# Supplementary material for: Regulation of photosynthesis, carbohydrate transport, and transcriptomic profiles in wheat by different fertilizers
Source: BMC Plant Biol. 2026 Jul 18;26:1767. doi: 10.1186/s12870-026-09506-6 (PMC13663238; doi:10.1186/s12870-026-09506-6)
Supplement: Supplementary file 1 [file 12870_2026_9506_MOESM1_ESM.docx]

**Supplementary Table** **1** Fertilizer dosage of each treatment in wheat-maize rotation system (kg·hm^−2^).

| **Season** | **Treatment** | **Urea** | **Double superphosphate** | **Potassium sulfate** | **[Pig manure](http://dict.cnki.net/dict_result.aspx?scw=%e7%8c%aa%e7%b2%aa%e5%a0%86%e8%82%a5&tjType=sentence&style=&t=pig+manure+compost)** | **Organic-inorganic compound fertilizer** |
| --- | --- | --- | --- | --- | --- | --- |
|  | CK | 652 | 261 | 200 | 0 | 0 |
| Wheat | T1 | 456 | 183 | 140 | 3600 | 0 |
|  | T2 | 0 | 0 | 0 | 0 | 3090 |
|  | CK | 544 | 90 | 90 | 0 | 0 |
| Maize | T1 | 380 | 63 | 63 | 3600 | 0 |
|  | T2 | 0 | 0 | 0 | 0 | 3090 |

**Supplementary Table 2** Nutrient profile of different fertilizers.

| **Fertilizer** | **N** | **P_2_O_5_** | **K_2_O** | **Organic matter** |
| --- | --- | --- | --- | --- |
| Pig manure | 1.63% | 2.51% | 1.01% | 38.89% |
| Organic-inorganic compound fertilizer | 9.96% | 2.44% | 1.59% | 29.38% |

**Supplementary Table 3** Nutrient inputs for each treatment (kg·hm^−2^).

|  | **Wheat season**  (N-P_2_O_5_-K_2_O) | **Maize season**  (N-P_2_O_5_-K_2_O) | **Annual nutrient input**  (N-P_2_O_5_-K_2_O) |
| --- | --- | --- | --- |
| CK | 300-120-100 | 250-45-45 | 860 (550-165-145) |
| T1 | 218-165-92 | 193-127.5-64.5 | 860 (411-292.5-156.5) |
| T2 | 308-74-48 | 308-74-48 | 860 (616-148-96) |

**Supplementary Figures 1.** PCA analysis of CK, T1 and T2 treatments.

 **Supplementary Figures 2.** Clustering heatmap of CK, T1 and T2 treatments.

**Supplementary Figures 3.** Correlation analysis between RNA-seq and qRT-PCR results of two datasets. CK1, T1-1, T2-1 were qRT-PCR results; CK2, T1-2, T2-2 were RNA-seq results.
